# Rumen Ciliated Protozoa of the Free-Living European Bison (*Bison bonasus*, Linnaeus)

**DOI:** 10.3389/fmicb.2021.658448

**Published:** 2021-06-28

**Authors:** Svetlana Kišidayová, Dominik Durkaj, Katarína Mihaliková, Zora Váradyová, Julia Puchalska, Małgorzata Szumacher-Strabel, Adam Cieślak, Zygmunt Gizejewski

**Affiliations:** ^1^Institute of Animal Physiology, Centre of Biosciences, Slovak Academy of Sciences, Košice, Slovakia; ^2^Institute of Biology and Ecology, Faculty of Science, Pavol Jozef Šafárik University, Košice, Slovakia; ^3^Department of Animal Nutrition, Poznan University of Life Sciences, Poznan, Poland; ^4^Department of Biodiversity Protection, Institute of Animal Reproduction and Food Research, Polish Academy of Sciences, Olsztyn, Poland

**Keywords:** *Bison bonasus*, ciliates, population diversity, protozoa, rumen

## Abstract

This study aims to perform population analysis of the rumen ciliated protozoa of the free-living European bison (wisent, *Bison bonasus*, Linnaeus). The samples of the rumen fluid from the 18 bison subjected to the controlled culls within the free-ranging population in the Bialowieza primeval forest in Poland were collected and examined. The examined ciliates population consisted of the species of the families *Isotrichidae* and *Ophryoscolecidae*. There were 12 genera (*Isotricha, Dasytricha, Diplodinium, Elytroplastron, Entodinium, Eodinium, Epidinium, Eremoplastron, Eudiplodinium, Metadinium, Ophryoscolex*, and *Ostracodinium*) and 32 morphospecies of the ciliates. We observed the prevalence of a type B protozoan population (56% animals) with the typical *Epidinium* and *Eudiplodinium* genera members. Other examined animals possessed the mixed A–B population with *Ophryoscolex* genus, distinct for type A ciliate population. The average total ciliates count was 2.77 ± 1.03 × 10^5^/ml (mean ± SD). The most abundant genera were *Entodinium*, 83%, and *Dasytricha*, 14%. The abundance of other genera was <1% of the total count. Within the 16 *Entodinium* species determined, the most abundant species was *Entodinium nanellum* (16.3% of total ciliates count). The average Shannon–Wiener diversity index was 2.1 ± 0.39, evenness was 0.7 ± 0.11, and species richness was 24 ± 3.0 (mean ± SD). Our study is the first report on the population composition and diversity of rumen ciliates of European bison. The composition and counts of ciliate genera and species were similar to the composition and counts of the rumen ciliated protozoa of American bison and many other kinds of free-living and domestic ruminants. Our European bison ciliate population analysis has shown medium ciliate density and high diversity typical for large free-living ruminants with mixed feeding behavior.

## Introduction

The European bison (wisent, *Bison bonasus* L.) is the largest terrestrial mammal in Europe. The European bison has been successfully restored after the extinction in the wild at the beginning of the twentieth century. The total area of European bison habitat covers about 130,000 ha in Poland. Due to herd management of growing free-ranging wisent population in the Bialowieza Forest (northeast Poland), the annual culls have been conducted since 1971 (Krasińska and Krasiński, 2013). They aim to reduce the population on average by 11%. The most frequent reasons for bison selections were various injuries, entering fields, aggression toward people, and, after 1980, changes in the genitourinary system caused by posthitis/balanoposthitis illness (Krasińska and Krasiński, 2013). The free-living ruminants are interesting from a scientific perspective because few studies describe their complex rumen microbial ecosystems (Ishaq et al., 2015). The complex rumen microbial population comprises prokaryotes (eubacteria and archaea) and eukaryotes (fungi and protozoa). The dominant component of rumen protozoa is ciliated protozoa (Williams and Coleman, 1992), which contribute to the breakdown of plant and microbial carbohydrates and proteins. Protozoa influence the rumen microbial population by predation on other protozoa, prokaryotes, and fungi, suggesting that protozoa are still attractive from a scientific point of view (Williams et al., 2020). In general, the number of rumen ciliates species in different host animals is about 45 or less, influenced mainly by host feeding behavior and seasonal variation in the diet (Dehority, 2004). The studies on the rumen ciliates population revealed two main types, types A and B. Type A population is characterized by the presence of genera of *Polyplastron* and *Ophryoscolex* (Eadie, 1962, 1967). Type B population is characterized by the presence of genera of *Epidinium* and *Eudiplodinium*. On the other hand, some other genera are commonly present in most ruminants (*Entodinium* spp. and *Isotrichids*). Rumen ciliates were described in many ruminants' species; however, their description of the genus *Bison* is limited only to American bison (A. bison, *Bison bison*). All protozoan species found in A. bison have also been reported in domestic livestock. The type B population predominated in free-living A. bison without contacts with cattle. The mixed A–B population occurred in bison in areas inhabited by domestic livestock (Towne et al., 1988b). The percentage distribution of rumen ciliate species among A. bison varies among the geographical regions, depending on the type and quantity of consumed feed and on the contact with other animals in the group. The present study aimed to examine and describe the ciliate population of the rumen fluid of European bison obtained from culled animals of the free-ranging population in Bialowieza. The description of rumen ciliate protozoa of the European bison contributes to our knowledge of the ecology and diversity of rumen ciliates of the free-living European ruminants.

## Materials and Methods

The material was obtained from culled European bison originating from the free-ranging population in the Bialowieza Forest (northeast Poland; longitude between 23°31′ and 24°21′ E and latitude 52°29′ and 52°57′ N). Samples were collected immediately after death (1–3 h) from 18 animals of both sexes of various ages during February and March 2007 (Table 1). For the microscopic counts, about 10 g/animal of the rumen contents were preserved with an equal amount of 8% formaldehyde solution (w/w), strained through four layers of cheesecloths into 10 ml polypropylene tubes with screw cups and stored at 8°C in a refrigerator until analysis. Ciliates were counted microscopically in an aliquot of the suitably diluted sample (Williams and Coleman, 1992). At least four replicates were counted per sample and per ciliate species. The protozoan genera and species were identified according to the size and the shape of cells, skeletal plates (if present), macronucleus, and ciliature arrangement (Dogiel, 1927; Ogimoto and Imai, 1981; Williams and Coleman, 1992; Ito and Imai, 1998; Ito et al., 2001; Cedrola et al., 2017a,b, 2018). Different staining procedures were used to stain skeletal plates (iodine solution), nuclei (methyl green-formalin-saline and chrome-alum-carmine), and infraciliature (pyridinated silver carbonate method) (Ogimoto and Imai, 1981). The pictures of ciliates were taken under bright field illumination by Moticam Pro CCD Camera (Motic Incorporation Ltd., Hong Kong) mounted on a BA400 microscope (Motic Incorporation Ltd., Hong Kong). The images were processed and analyzed using ImageJ software according to ImageJ software documentation (Abramoff et al., 2004; Siritantikorn et al., 2012; Choudhry, 2016). Morphometric measurements were performed by image analysis of at least 20 cells. Only ciliates of the families Isotrichidae and Ophryoscolecidae were present in the samples. Differentiated count of species of genus *Entodinium* was performed in 13 samples (animals). Direct bacterial count estimated the total bacteria count through image analysis of pictures taken under bright field illumination of dried smears of formaldehyde-fixed samples (Siritantikorn et al., 2012; Choudhry, 2016). Two smears stained with methylene blue and known dimensions and known volumes per sample (animal) were prepared according to the Breed method (Horáková, 1988). Twenty randomly selected pictures per smear were taken at an objective magnification of ×100 by the Moticam Pro CCD Camera (Motic Incorporation Ltd., Hong Kong) mounted on a BA400 microscope (Motic Incorporation Ltd., Hong Kong). The images were processed and analyzed using ImageJ software (Selinummi et al., 2005). Ciliates and bacteria counts per milliliter were expressed as geometric means of log natural transformed values ± geometric standard deviation and arithmetic means ± standard deviations. We evaluated bison age and sex effects on the total count of bacteria and ciliates by nonparametric Kruskal–Wallis test (GraphPad Prism, GraphPad Software, Inc., San Diego, CA, USA). The biodiversity indices (Shannon–Wiener diversity index, evenness, and species richness) (Spellerberg and Fedor, 2003; Jost, 2006) were calculated with an Excel calculator (Microsoft Office Professional, 2007, Microsoft, Redmond, WA, USA). The Shannon–Wiener (SW) diversity index was computed to explain the entropy, taking into account the species richness and evenness of the community. The species richness was evaluated by counting the number of taxa per sample (animal). Correlation analysis on counts of ciliates genera was calculated with Prism 5 (GraphPad Software, Inc., San Diego, CA, USA). Probability value *p* < 0.05 was considered significant. Principal component analysis was performed on counts of ciliates genera with the aid of the STATISTICA (Data Analysis Software System), version 9.0. (StatSoft, Inc., Tulsa, OK, USA, 2009).

**Table 1:** Average weight, age, rumen ciliates, and bacteria counts of European bison (wisent, *Bison bonasus*, L.).

	**Bison counts**	**Weight (kg)**		**Age (year)**		**Ciliate count (Ln/ml)**		**Bacteria count (Ln/ml)**	
	***n***	**Mean**	**SD**	**Mean**	**SD**	**Geom-mean**	**Geom-SD**	**Geom-mean**	**Geom-SD**
Males	5	309	92.3	2.6	0.89	12.32	1.714	23.81	1.410
Females	6	395	73.1	12.4	8.32	12.25	1.679	23.81	1.254
Young females	7	108	16.1	0.6	0.18	12.34	2.498	23.92	1.168

## Results

**Figure 1:**
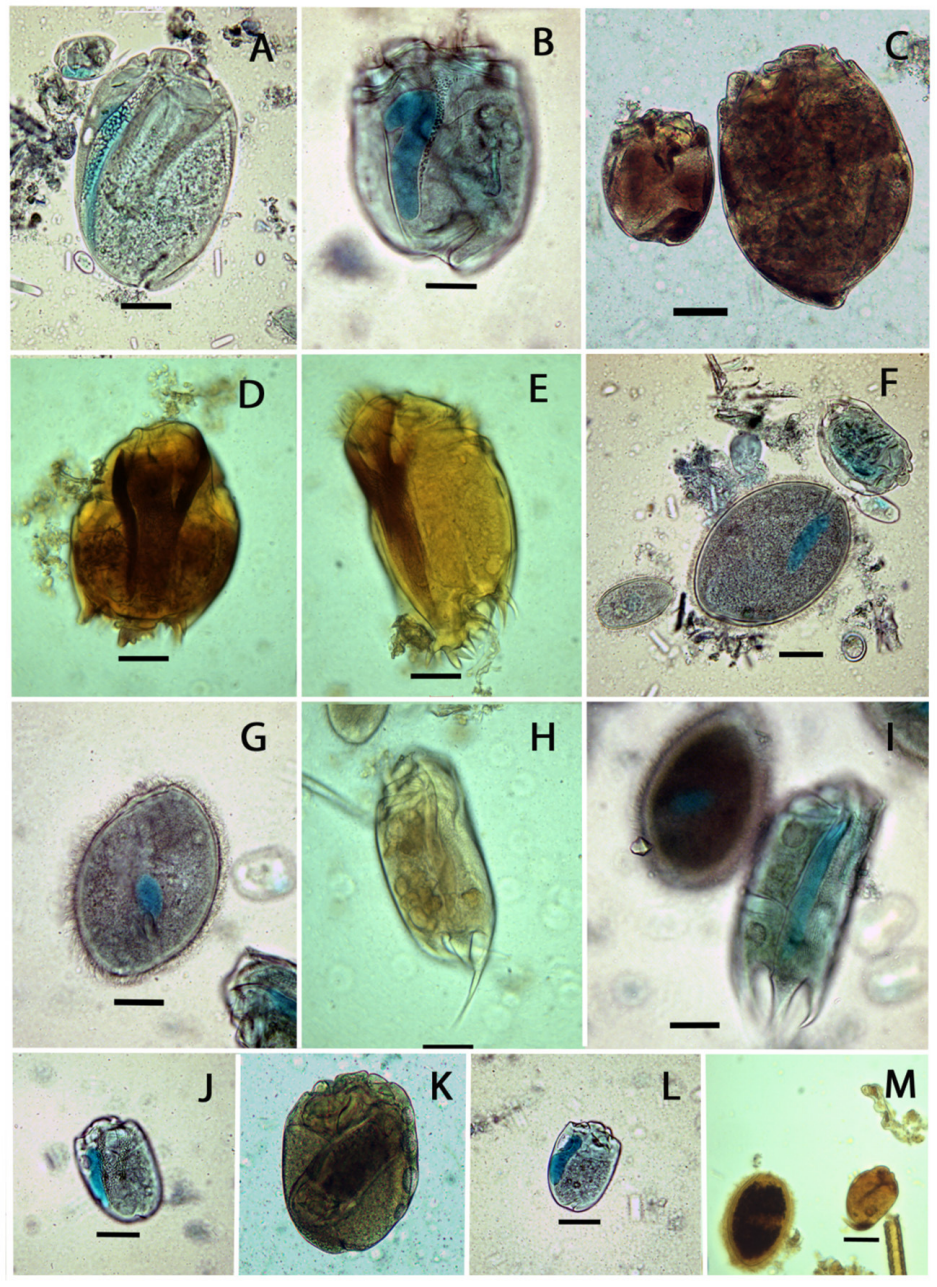
Photomicrographs of some ciliates observed in the rumen of European bison. **(A)** *Elytroplastron bubali*; **(B)** *Eudiplodinium maggii*; **(C)** the illustration of two extreme sizes of *E. maggii*; **(D**,**E)** *Ophryoscolex purkyniei*; **(F)** (from left to right) *Dasytricha ruminantium, Isotricha prostoma, Diplodinium dentatum*, and *Entodinium exiguum*; **(G**,**I)** *Isotricha intestinalis*; **(H**,**I)** *Epidinium parvicaudatum*; **(J)** *Metadinium esalqum*; **(K)** *Elytroplastron* with engulfed *Epidinium*; **(L)** *Eodinium posterovesiculatum*; **(M)** *Dasytricha ruminantium* and *Eremoplastron rostratum*. Samples were colorized by **(A**,**B**,**F**,**G**,**I–L)** methyl-green formalin, **(D**,**E**,**H**,**M)** iodine solutions, and **(C)** pyridinated silver carbonate. Scale bars are 20 μm in all pictures.

**Figure 2:**
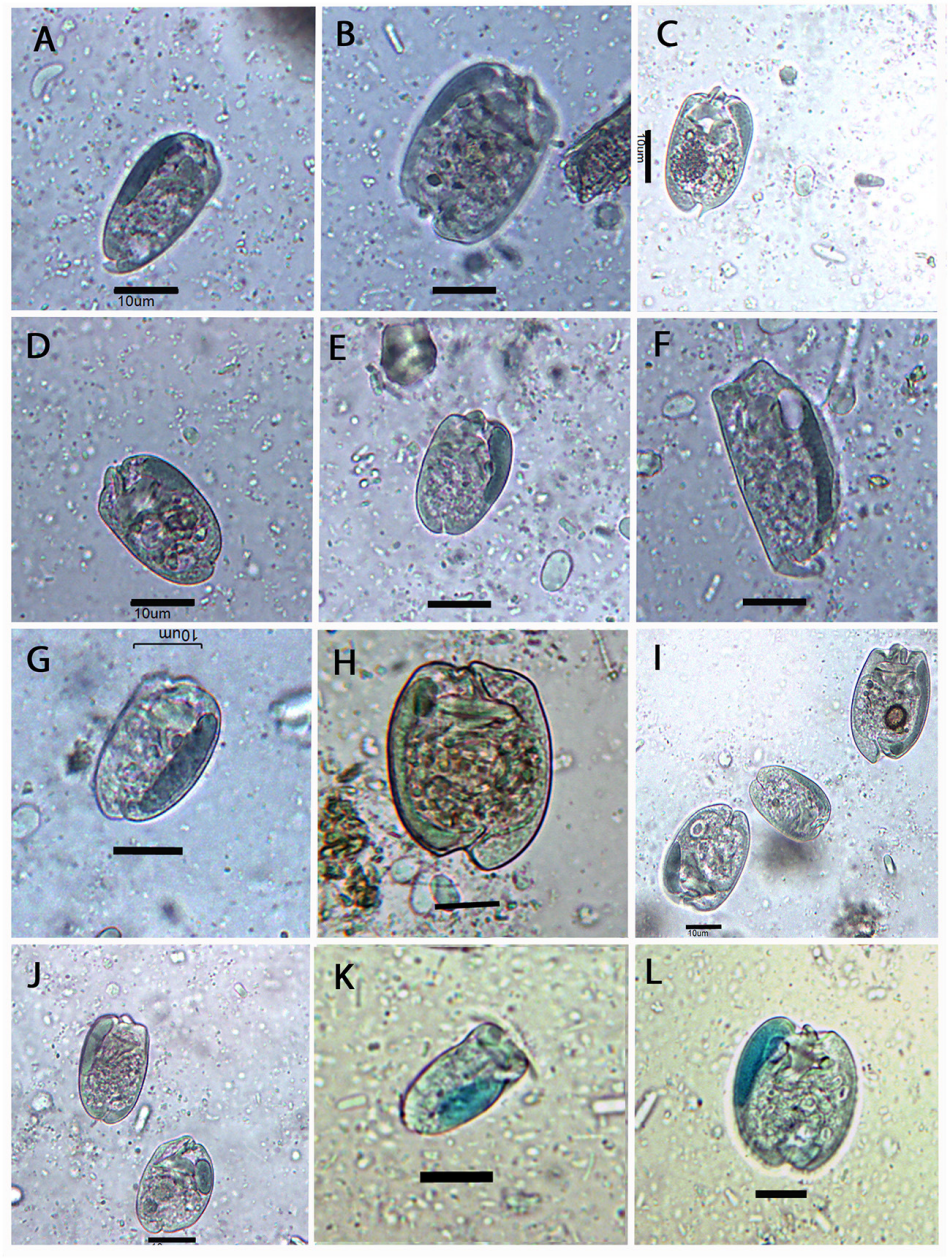
Photomicrographs of some entodiniid ciliates observed in the rumen of European bison. **(A)** *Entodinium brevispinum*; **(B)** *E. caudatum*; **(C)** *E. lobosospinosum*; **(D)** *E. simplex*; **(E)** *E. nanellum*; **(F)** *E. rostratum*; **(G)** *E. parvum*; **(H)** *E. yunense*; **(I)** (from left to right) *E. simplex, E. nanellum*, and *E. yunense*; **(J)** *E. orbicularis*; **(K)** *E. exiguum*; and **(L)** *E. dubardi*. Samples were colorized by **(A–J)** chrome-alum-carmine and **(K,L)** methyl-green formalin solutions. Scale bars are 10 μm in all pictures.

**Figure 3:**
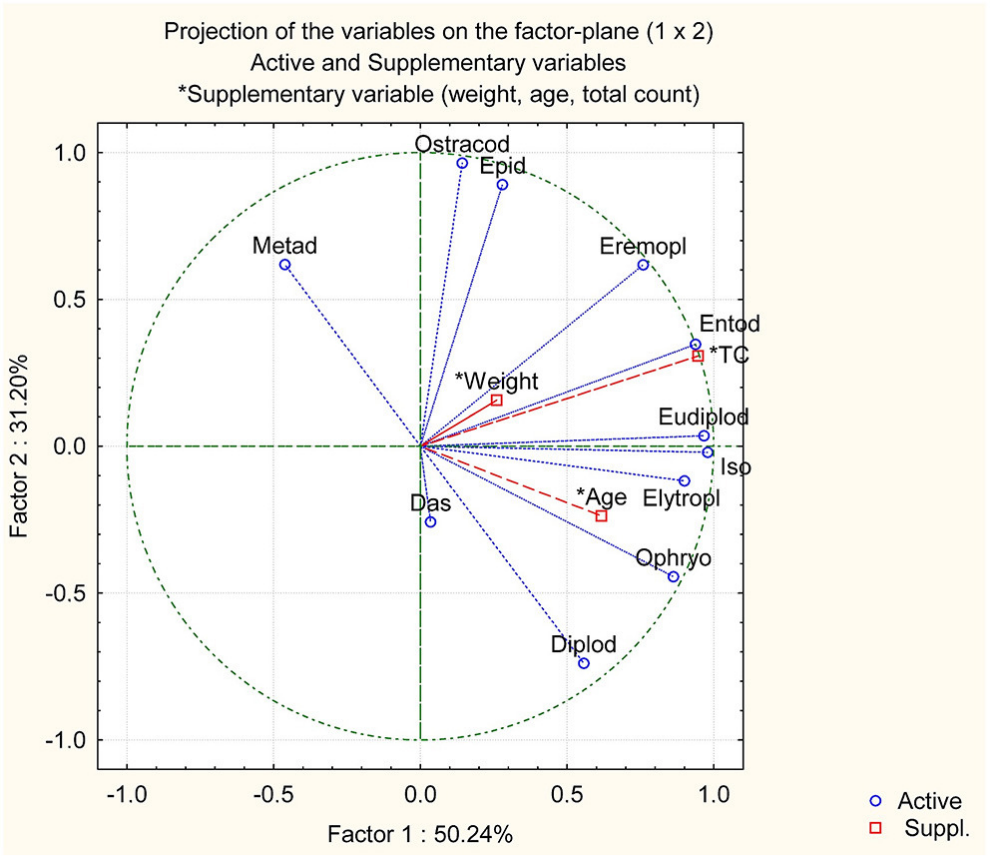
Principal component analysis of the European bison ciliates population. *Metadinium* (Metad), *Ostracodinium* (Ostracod), *Epidinium* (Epid), *Eremoplastron* (Eremopl), *Entodinium* (Entod), total count (TC), *Eudiplodinium* (Eudiplod), *Isotricha* (Iso), *Elytroplastron* (Elytropl), *Ophryoscolex* (Ophryo), *Diplodinium* (Diplod), and *Dasytricha* (Das).

Age, weight, and sex of hosts and the total count of ciliates and bacteria are summarized in Table 1. The abundance of bacteria was estimated to be 235.10^8^ ± 43 × 10^8^/ml (arithmetic mean ± SD). The mean count of ciliates was 2.77 × 10^5^ ± 1.03 × 10^5^/ml (arithmetic mean ± SD). We observed no effects of sex and age of host on the abundance of bacteria (*P* = 0.809) and ciliates (*P* = 0.412). We determined 12 genera and 32 ciliates species by microscopic examination (Tables 2, 3). All examined bison ciliates populations can be classified as type B, although the *Ophryoscolex* genus was present in eight individuals. We observed the following genera and morphospecies of the family Isotrichidae: *Isotricha* (*Isotricha prostoma, Isotricha intestinalis*) and *Dasytricha* (*Dasytricha ruminantium*). We observed the following genera and morphospecies of the family Ophryoscolecidae: *Diplodinium* (*Diplodinium dentatum*), *Entodinium* (*Entodinium brevispinum, Entodinium caudatum, Entodinium dubardi, Entodinium exiguum, Entodinium furca, Entodinium lobosospinosum, Entodinium longinucleatum, Entodinium nanellum, Entodinium nanum, Entodinium ovinum, Entodinium ovoideum, Entodinium parvum, Entodinium rostratum, Entodinium simplex, Entodinium triacum*, and *Entodinium yunnense*), *Elytroplastron* (*Elytroplastron bubali*), *Eodinium* (*Eodinium posterovesiculatum*), *Epidinium* (*Epidinium caudatum, Epidinium ecaudatum, Epidinium parvicaudatum, Epidinium quadricaudatum*, and *Epidinium tricaudatum*), *Eremoplastron* (*Eremoplastron rostratum*), *Eudiplodinium* (*Eudiplodinium maggii*), *Metadinium* (*Metadinium esalqum*), *Ostracodinium* (*Ostracodinium gracile*), and *Ophryoscolex* (*Ophryoscolex purkyniei*), (Figures 1, 2). The most abundant genera were *Entodinium* (83%, 2.22 × 10^5^/ml) and *Dasytricha* (13%, 0.33 × 10^5^/ml). The remaining genera were <1% abundant. The genera of *Entodinium, Epidinium, Diplodinium, Eudiplodinium, Elytroplastron*, and *Dasytricha* were present in all rumen samples. The *E. posterovesiculatum* was infrequent and not countable. The *Epidinium* species of *E. caudatum, E. ecaudatum, E. quadricaudatum*, and *E. tricaudatum* were infrequent and not countable. The dominant *Epidinium* species was *E. parvicaudatum*. Within other large ciliates, the least abundant species were *E. bubali* and *O. gracile*. Within the *Entodinium* species, the most numerous were *E. nanellum*, with 16.3% of total ciliates count (Table 3). Other *Entodinium* species were abundant <5% of the total ciliates count. Table 4 shows the evaluation of the population variability of rumen ciliates of European bison with diversity (Shannon–Wiener index) of 2.1, evenness (Peliou index) of 0.7, and species richness of 24. Correlation analysis is summarized in Table 5 (the numbers within brackets are r and P, respectively). Analysis revealed the positive correlation of *Isotricha* counts with counts of *Entodinium* (0.73, *0.001*), total counts (0.72, *0.001*), *Ostracodinium* (0.68, *0.02*), *Eudiplodinium* (0.63, *0.007*), *Eremoplastron* (0.66, *0.01*), and *Elytroplastron* (0.62, *0.008*). *Entodinium* counts correlated positively with total counts (0.94, *0.001*), counts of *Eremoplastron* (0.83, *0.001*), *Isotricha* (0.73, *0.01*), *Eudiplodinium* (0.72, *0.01*), and *Ostracodinium* (0.62, *0.04*). *Epidinium* counts correlated positively with counts of *Eremoplastron* (0.66, *0.01*) and negatively with animal weight (−0.47, *0.05*). *Eremoplastron* counts correlated with counts of *Entodinium* (0.83, *0.001*), totals (0.78, *0.002*), *Eudiplodinium* (0.75, *0.003*), *Ostracodinium* (0.75, *0.03*), *Epidinium* (0.66, *0.01*), and *Isotricha* (0.66, *0.01*). *Eudiplodinium* counts correlated with counts of *Eremoplastron* (0.75, *0.003*), *Entodinium* (0.72, *0.001*), *Isotricha* (0.63, *0.01*), and totals (0.63, *0.005*). *Elytroplastron* counts correlated with the counts of *Ostracodinium* (0.93, *0.001*) and *Isotricha* (0.62, *0.008*). *Ophryoscolex* counts correlated only with animal age (0.73, *0.04*). *Ostracodinium* counts correlated with the counts of *Elytroplastron* (0.93, *0.001*), *Eremoplastron* (0.75, *0.03*), *Isotricha* (0.68, *0.02*), and *Entodinium* (0.62, *0.04*). No significant correlations were observed on counts of *Dasytricha, Diplodinium*, and *Metadinium*. No correlations were observed on animal gender. Animal weight was associated with animal age (0.70, *0.001*). These relationships are illustrated with a PCoA plot of the variables, which shows similar relationships (Figure 3).

**Table 2:** The analysis of rumen ciliated protozoa population of European bison (*Bison bonasus*. L.).

**Family, genus, species**	**Prevalence (%)**	**Percentage of total count(%)**	**Count (C/ml)**					
			**Mean**	**SD**	***n***	**Median**	**Min**	**Max**
*Ophryoscolecidae*								
*Entodinium* spp.	100	82.86	229,537	94,228.29	18	217,333	90,666	404,000
*Epidinium*								
*Epidinium parvicaudatum*	100	0.29	812	1,116.28	18	402	13	4,870
*Eremoplastron*								
*Eremoplastron rostratum*	72	0.30	831	1,188.05	13	350	100	3,950
*Diplodinium*								
*Diplodinium dentatum*	100	0.86	2,390	1,578.42	18	2,120	13	5,180
*Metadinium*								
*Metadinium esalqum*	100	0.13	366	331.67	18	264	60	1,307
*Eudiplodinium*								
*Eudiplodinium maggii*	100	0.23	636	598.50	18	310	13	2,040
*Elytroplastron*								
*Elytroplastron bubali*	100	0.12	345	409.64	18	270	7	1,727
*Ostracodinium*								
*Ostracodinium gracile*	61	0.01	32	44.21	11	20	7	160
*Ophryoscolex*								
*Ophryoscolex purkynei*	44	0.03	92	135.33	8	30	5	400
*Isotrichidae*								
*Dasytricha*								
*Dasytricha ruminantium*	100	14.36	39,787	33,637.57	18	42,800	400	144,000
*Isotricha*								
*Isotricha prostoma*	78	0.48	1,317	1,399.83	14	707	253	4,013
*Isotricha intestinalis*	83	0.39	1,071	1,543.91	15	440	40	5,520
Total count	100	100	277,022	103,503.93	18	274,620	105,279	463,181

**Table 3:** The counts of rumen *Entodinium* species of European bison (*Bison bonasus*. L.).

**Species**	**Prevalence %**	**Percentage of total count %**	**Count C/ml**					
			**Mean**	**SD**	***n***	**Median**	**Min**	**Max**
*E. brevispinum*	92	2.6	7,300	5,345	12	5,000	400	17,200
*E. caudatum*	77	0.7	1,880	1,544	10	1,600	200	4,800
*E. dubardi*	100	3.5	9,660	6,144	13	9,200	1,000	19,200
*E. exiguum*	100	4.6	12,740	8,060	13	9,600	3,800	32,000
*E. furca monolobum*	92	1.6	4,430	2,885	12	3,800	200	8,800
*E. lobosospinosum*	92	1.4	4,020	2,680	12	3,900	600	9,400
*E. longinucleatum*	100	1.3	3,520	2,385	13	3,600	1,000	8,200
*E. nanellum*	100	16.3	45,060	25,686	13	41,800	11,800	95,800
*E. nanum*	100	5.0	13,862	7,744	13	12,800	3,600	28,200
*E. ovinum*	8	0.1	200		1	200	200	200
*E. ovoideum*	92	2.7	7,450	4,408	12	6,500	2,000	17,000
*E. parvum*	100	4.6	12,750	7,683	13	11,400	800	23,800
*E. rostratum*	77	1.4	3,980	6,275	10	1,900	400	21,600
*E. simplex*	100	1.7	4,830	3,513	13	4,200	200	11,600
*E. triacum*	85	1.3	3,530	3,501	11	1,400	200	11,200
*E. yunnense*	92	0.3	820	679	12	600	200	2,000

**Table 4:** Evaluation of population variability of rumen ciliates of European bison.

	**Mean**	**SD**	**Median**	**Min**	**Max**	***n***
Diversity (SW index)	2.1	0.39	2.3	1.4	2.6	13
Evenness (Peliou index)	0.7	0.11	0.7	0.5	0.8	13
Species richness	24	3.0	24	15	27	13

**Table 5:** Correlation matrix of animal gender, age, weight, and rumen ciliate counts of European bison.

	**Gender**	**Age, years**	**Weight, kg**	**Entod**	**Epid**	**Eremopl**.	**Eudipl**.	**Elytropl**.	**Metad**.	**Ophryo**.	**Ostracod**.	**Dasytr**.	**Isotr**.	**Diplod**.
Gender														
Age, years	0.32 (0.20)													
Weight, kg	−0.11 (0.67)	**0.70 (0.001)**												
Entod	−0.03 (0.90)	−0.13 (0.62)	−0.25 (0.32)											
Epid	0.15 (0.56)	−0.33 (0.19)	**−0.47 (0.05)**	0.02 (0.93)										
Eremo	−0.12 (0.69)	0.19 (0.54)	0.33 (0.27)	**0.83 (0.001)**	**0.66 (0.01)**									
Eudipl	0.07 (0.77)	0.14 (0.59)	0.14 (0.58)	**0.72 (0.001)**	0.74 (0.64)	**0.75 (0.003)**								
Elytropl	0.39 (0.11)	−0.14 (0.57)	−0.36 (0.15)	0.40 (0.11)	0.23 (0.35)	0.25 (0.40)	0.10 (0.68)							
Metad	0.10 (0.69)	−0.08 (0.76)	−0.07 (0.78)	0.22 (0.38)	−0.01 (0.97)	0.29 (0.34)	−0.04 (0.86)	0.03 (0.92)						
Ophryo	0.30 (0.47)	**0.73 (0.04)**	0.43 (0.29)	0.33 (0.43)	−0.38 (0.36)	0.36 (0.43)	0.69 (0.06)	0.59 (0.13)	−0.35 (0.39)					
Ostrac	0.20 (0.55)	−0.27 (0.42)	−0.40 (0.22)	**0.62 (0.04)**	0.03 (0.94)	**0.75 (0.03)**	0.17 (0.63)	**0.93 (0.001)**	0.02 (0.95)	−0.34 (0.51)				
Dasytr	−0.26 (0.30)	−0.07 (0.78)	0.11 (0.67)	−0.24 (0.95)	−0.24 (0.33)	0.02 (0.94)	−0.16 (0.52)	−0.12 (0.64)	0.20 (0.44)	−0.10 (0.82)	−0.11 (0.75)			
Isotr	0.30 (0.24)	0.08 (0.77)	−0.37 (0.15)	**0.73 (0.001)**	0.40 (0.11)	**0.66 (0.01)**	**0.63 (0.007)**	**0.62 (0.008)**	−0.03 (0.92)	0.34 (0.41)	**0.68 (0.02)**	0.01 (0.97)		
Diplod	0.03 (0.90)	0.04 (0.32)	0.04 (0.88)	0.46 (0.05)	−0.13 (0.62)	0.32 (0.29)	0.25 (0.31)	−0.02 (0.94)	0.14 (0.59)	0.49 (0.22)	−0.14 (0.69)	0.33 (0.18)	0.41 (0.10)	
Totals	−0.10 (0.69)	−0.13 (0.61)	−0.20 (0.43)	**0.94 (0.001)**	−0.05 (0.84)	**0.78 (0.002)**	**0.63 (0.005)**	0.34 (0.17)	0.28 (0.27)	0.29 (0.48)	0.59 (0.06)	0.32 (0.20)	**0.72 (0.001)**	**0.56 (0.02)**

*The first value is correlation coefficient r, and a number in parentheses is probability value P. The significant values are highlighted*.

## Discussion

The rumen protozoal population of free-living ruminants is influenced mainly by host feeding behavior and seasonal variation in the diet (Kamler, 1999; Dehority and Odenyo, 2003; Booyse and Dehority, 2011; Clauss et al., 2011; Obidziński et al., 2017). European bison can be considered mixed feeders with 68–97% of herbaceous plants in their natural diet (Gebczyńska et al., 1991; Kowalczyk et al., 2019). In some other references, the European bison are considered as grazers with 68% of grass in their natural diet (Pucek et al., 2002; Clauss et al., 2006; Przybyło et al., 2019). The population of rumen ciliated protozoa of grazers (e.g., cattle) is generally more diverse in comparison with typical browsers, selectors (e.g., roe deer and blue duiker), but this is not a rule (Kofoid and Christianson, 1934; Sládeček, 1946; Prins and Geelen, 1971; Giesecke and Gylswyk, 1975; Imai, 1988; Williams and Coleman, 1992; Dehority, 1994; Robbins et al., 1995; Dehority and Odenyo, 2003; Clauss et al., 2011). More generically diverse ciliate populations of grass and roughage eaters may result from a slower passage rate and higher rumen pH (Dehority and Odenyo, 2003). However, in the Bialowieza forest, other food (hay) is available to bison during winter (Gebczyńska et al., 1991; Pucek et al., 2002; Kowalczyk et al., 2011). Therefore, the differences in both summer and winter rumen ciliate populations are likely small. To our knowledge, this is the first study describing the population of protozoa in the rumen of European bison of the Białowieza region. Generally, the species composition of rumen ciliates of European bison was similar to the composition of rumen ciliates populations of many other species of free-living and domesticated ruminants (Kofoid and Christianson, 1934; Sládeček, 1946; Crha, 1972; Towne et al., 1988a,b; Ito et al., 1994; Moon-van der Staay et al., 2014). Recent research pointed to the existence of a core rumen microbiome across a wide geographical range, which is modified by the diet and the host (O'Kelly and Spiers, 1992; Guan et al., 2008; Shi et al., 2008; Moon-van der Staay et al., 2014; Henderson et al., 2015; Ishaq et al., 2015; Tapio et al., 2017; Reis et al., 2019; Furman et al., 2020; Xue et al., 2020). These studies indicate that dietary and animal feeding strategies dominate over host species. In the study of Henderson et al. (2015), the variability of protozoa between and within animal groups was much greater than that of bacteria and archaea. Analyses of different 18S ribosomal RNA (rRNA) genes showed extremely complex but related ciliate communities, which occur in the rumen of cattle, sheep, goats, and red deer (Moon-van der Staay et al., 2014). Although individual host genetic characteristics might influence the composition of the rumen prokaryotes (Shi et al., 2008; Xue et al., 2020), it seems that ciliates' feed preferences and relationship within ciliates contribute remarkably to their composition in the rumen (Eadie, 1967; Dehority, 1998; Martinele and D'Agosto, 2008). *In vitro* and *in vivo* experiments revealed the competitive relationship between certain starch preferring *Entodinium* species (*Entodinium caudatum*) and the fibrolytic ciliates *Eudiplodinium maggii* and *Epidinium ecaudatum* (Michalowski et al., 2003; Bełzecki et al., 2004; Zeitz et al., 2012). Both American bison and European bison have a similar ciliates genera composition with the prevalence of a type B protozoan population. Typical members of the type B population are *Epidinium, Eudiplodinium, Metadinium*, and *Elytroplastron*. On the other hand, typical antagonistic types A population members are *Polyplastron, Ophryoscolex*, and *Diploplastron* (Eadie, 1962, 1967). It is known that *Polyplastron multivesiculatum* grazes on *Epidinium*, resulting in the vanish of *Epidinium* from the rumen (Williams and Coleman, 1992). We noted 44% of animals with a mixed A–B population. The prevalence of type B protozoan population was observed in many species of free-living ruminants, e.g., deer, ibex, mouflon, chamois, moose, reindeer, muskox, gaur, antelopes, and giraffe (Kofoid and Christianson, 1934; Sládeček, 1946; Kleynhans and van Hoven, 1976; Kleynhans, 1982; Crha et al., 1985; Dehority, 1985, 1986; Dehority et al., 1999; Dehority and Odenyo, 2003; Karnati et al., 2003; Imai et al., 2004; Korchagina, 2006, 2012; de la Fuente et al., 2009). In contrast to A. bison, we observed only *Ophryoscolex* spp. (*O. purkyniei*) and no *Polyplastron* spp. in animals with mixed A–B protozoan population. Therefore, *Epidinium* species prevalence was not influenced in animals with mixed A–B protozoan populations (Table 2). Besides, the predatory behavior of rumen ciliates was observed not only in the type A population. In the type B population, the predatory behavior of *E. bubali* on *Epidinium, Enoploplastron*, and *Entodinium* was observed in sheep rumen (Martinele and D'Agosto, 2008). However, the predatory activity of *E. bubali* in our bison samples was low. Our correlation analysis revealed the prevalence of the positive correlations among the individual rumen protozoal genera counts. Most numerous positive correlations were observed on *Isotricha* spp. (with total counts, *Entodinium, Eremoplastron, Eudiplodinium, Elytroplastron*, and *Ostracodinium* counts). We can speculate that the growth of *Isotricha* can be promoted by soluble metabolic products of hydrolytic and proteolytic activities of *Entodinium, Eremoplastron, Eudiplodinium, Elytroplastron*, and *Ostracodinium* species. *Isotricha* is known to prefer soluble substrates (Williams, 1986). On the other hand, Entodiniomorphid ciliates prefer solid substrates (plant and bacterial particles) (Williams and Coleman, 1992). On the other hand, we have observed no correlations of *Dasytricha* and *Metadinium* with other ciliate genera. Differences in metabolic activities were observed between *Isotricha* and *Dasytricha*. They remarkably differ in carbohydrate fermentation (Howard, 1959). *Dasytricha* has greater metabolic versatility than *Isotricha*. Therefore, *Dasytricha* is probably less dependent on intermediate metabolic products of other members of the ciliate population. We can speculate on similar features of *Metadinium* species. For example, *Metadinium medium* could degrade starch, amylose, amylopectin, and hemicellulose (Naga and El-Shazly, 1968). The comparison with other studies is difficult because of different analysis methods, different animal diets, and different ciliate population structures observed. In Tan et al. (2020), the correlation of molecular data revealed the positive association of the *Metadinium* with *Eudiplodinium, Isotricha* with *Dasytricha*, and *Polyplastron* with *Ostracodinium* and *Ophryoscolex*. In our study, those associations were not observed. The influence of the season may also contribute to these differences. Our samples were taken in the winter when the animals eat a predominantly fibrous diet with a lack of green fodder rich in soluble nutrients. Our ciliate population analysis has shown medium ciliate density and high diversity typical for large free-living ruminants with mixed feeding behavior. We observed a similar total number of rumen ciliates of European bison (277.10^3^/ml) and American bison (328.10^3^/ml) (Towne et al., 1988a,b). A similar ciliate density was also observed in domestic ruminants (cattle and goats) (Imai, 1988; Ito et al., 1994, 1995; de la Fuente et al., 2009; Mishima et al., 2009). On the other hand, the domestic ruminants have the lower average number of ciliate species per host (8–18; Imai, 1988; Ito et al., 1994, 1995; de la Fuente et al., 2009; Mishima et al., 2009). It is considered that species evenness decreases in the ruminants on high concentrate feed (Ito et al., 1994). On the other hand, when ruminants are fed on high forage, the number of species per host increases to more than 30 (Mishima et al., 2009). There were considerable variations in the counts of all examined ciliates species (genera). The animal-to-animal variations in both the differential counts of ciliate species and total counts were also observed in A. bison and other ruminants (Towne et al., 1988a,b; Kittelmann and Janssen, 2011). Purser and Moir (Purser and Moir, 1966a,b) showed that rumen volume could be a factor involved in individual animal differences in rumen parameters of sheep fed the same diet. The significant differences in the total ciliates counts were removed after the counts' adjustment for rumen volume (Dehority, 1978). The same author observed decreased rumen volume of sheep fed concentrate diet than the forage (alfalfa) diet. The changes in the rumen volume regarding the feed changes were also observed in other ruminants (Kamler et al., 2003). Some studies also suggest the effects of animal age, sex, and weight on the ciliate population (Clauss et al., 2011; Duarte et al., 2018). Our correlation analysis revealed no effects of animal sex on ciliates counts. However, sex in our bison collection was not evenly represented among age groups. No effects of host age and sex on rumen protozoa were observed on Spanish ibex and domestic goats (de la Fuente et al., 2009). We have observed a positive correlation of *Ophryoscolex* counts with animal age and a negative correlation of *Epidinium* counts with host weight. Those relationships' physiological backgrounds are unclear, as analysis revealed the positive correlation of animal age and weight. It can point to the possible antagonistic relationship between *O. purkyniei* and *E. parvicaudatum*. This phenomenon needs more not only microscopic but also molecular and *in vitro* physiological studies.

## Conclusion

Our study is the first report on the population composition and diversity of rumen ciliates of European bison. The population structure and counts of ciliate genera and species of European bison were similar to the composition and counts of the rumen ciliated protozoa of American bison and many other kinds of free-living and domestic ruminants. Our European bison ciliate population analysis has shown medium ciliate density and high diversity typical for large free-living ruminants with mixed feeding behavior.

## Data Availability Statement

The original contributions presented in the study are included in the article/supplementary material, further inquiries can be directed to the corresponding authors.

## Ethics Statement

The animal study was reviewed and approved by the Ethical Committee of the Institute of Animal Physiology of Centre of Biosciences of SAS approved the experimental protocol (resolution number Ro-3355/16-221).

## Author Contributions

SK: project administration, investigation and methodology, validation, samples analysis and evaluation, data curation, manuscript writing, review, and editing. DD and KM: samples analysis and evaluation. ZV: investigation and methodology and manuscript review. JP: samples collection. MS-S: funding acquisition and supervision and manuscript review. AC: conceptualization, funding acquisition and supervision, and manuscript review. ZG: conceptualization, samples collection, and manuscript review. All authors read and approved the final manuscript.

## Conflict of Interest

The authors declare that the research was conducted in the absence of any commercial or financial relationships that could be construed as a potential conflict of interest.

## References

[B1] Abramoff M. D. Magalhães P. J. Ram S. J. (2004). Image processing with ImageJ. Biophotonics Int. 11, 36–42.

[B2] Bełzecki G. Miltko R. Michalowski T. (2004). Why does the establishment of the starch preferring *Entodinium caudatum* in the rumen decrease the numbers of the fibrolytic ciliate *Eudiplodinium maggii*?. Folia Microbiol. 49, 139–142. 10.1007/BF02931388 15227784

[B3] Booyse D. Dehority B. A. (2011). Protozoa and digestive tract parameters of the impala. Onderstepoort J. Vet. Res. 78:327. 10.4102/ojvr.v78i1.327 23327216

[B4] Cedrola F. Dias R. J. P. Martinele I. D'Agosto M. (2017a). Polymorphism and inconsistencies in the taxonomy of *Diplodinium anisacanthum* da Cunha, 1914 (Ciliophora, Entodiniomorphida, Ophryoscolecidae) and taxonomic notes on the genus *Diplodinium* Schuberg, 1888. Zootaxa 4306:249. 10.11646/zootaxa.4306.2.5

[B5] Cedrola F. Rossi M. F. Martinele I. D'Agosto M. Dias R. Júnio P.. (2018). Morphology and description of infraciliary bands pattern in four *Metadinium Awerinzew*; *Mutafowa*, 1914 species *(Ciliophora, Entodiniomorphida, Ophryoscolecidae*) with taxonomic notes on the genus. Zootaxa 4500:574. 10.11646/zootaxa.4500.4.6 30486051

[B6] Cedrola F. Senra M. V. X. D'Agosto M. Dias R. J. P. (2017b). Phylogenetic analyses support validity of genus *Eodinium* (*Ciliophora, Entodiniomorphida, Ophryoscolecidae*). J. Eukaryot. Microbiol. 64, 242–247. 10.1111/jeu.12355 27539116

[B7] Choudhry P. (2016). High-throughput method for automated colony and cell counting by digital image analysis based on edge detection. PLoS ONE 11:e0148469. 10.1371/journal.pone.0148469 26848849 PMC4746068

[B8] Clauss M. Hofmann R. R. Hummel J. Adamczewski J. Nygren K. Pitra C.. (2006). Macroscopic anatomy of the omasum of free-ranging moose (*Alces alces*) and muskoxen (*Ovibos moschatus*) and a comparison of the omasal laminal surface area in 34 ruminant species. J. Zool. 270, 346–358. 10.1111/j.1469-7998.2006.00148.x

[B9] Clauss M. Müller K. Fickel J. Streich W. J. Hatt J.-M. Südekum K.-H. (2011). Macroecology of the host determines microecology of endobionts: protozoal faunas vary with wild ruminant feeding type and body mass. J. Zool. 283, 169–185. 10.1111/j.1469-7998.2010.00759.x

[B10] Crha J. (1972). Rumen ciliates in Fallow Deer (*Dama Dama L*.) in Namest preserve. Acta Vet. Brno 41, 355–362.

[B11] Crha J. Hrabě V. Koubek P. (1985). Rumen ciliate fauna in the chamois (*Rupicapra rupicapra L*.). Acta Vet. Brno 54, 141–147. 10.2754/avb198554030141

[B12] de la Fuente G. Belanche A. Abecia L. Dehority B. A. Fondevila M. (2009). Rumen protozoal diversity in the Spanish ibex (*Capra pyrenaica hispanica*) as compared with domestic goats (*Capra hircus*). Eur. J. Protistol. 45, 112–120. 10.1016/j.ejop.2008.07.001 18929470

[B13] Dehority B. A. (1978). Specificity of rumen ciliate protozoa in cattle and sheep^*^. J. Protozool. 25, 509–513. 10.1111/j.1550-7408.1978.tb04177.x

[B14] Dehority B. A. (1985). Rumen ciliates of musk-oxen (*Ovibos moschatus Z*.) from the canadian arctic. J. Protozool. 32, 246–250. 10.1111/j.1550-7408.1985.tb03045.x

[B15] Dehority B. A. (1986). “Microbes in the foregut of arctic ruminants,” in Control of Digestion and Metabolism in Ruminants, eds L. P. Milligan, W. L. Grovum, and A. Dobson (Prentice-Hall Englewood Cliffs), 307–325.

[B16] Dehority B. A. (1994). Rumen ciliate protozoa of the blue duiker (*Cephalophus monticola*), with observations on morphological variation lines within the species *Entodinium dubardi*. J. Eukaryot. Microbiol. 41, 103–111. 10.1111/j.1550-7408.1994.tb01481.x 8167616

[B17] Dehority B. A. (1998). Microbial interactions in the rumen. Rev. Fac. Agron. 69–86.

[B18] Dehority B. A. (2004). Rumen Microbiology. Thrumpton: Nottingham University Press.

[B19] Dehority B. A. Demarais S. Osborn D. A. (1999). Rumen ciliates of white-tailed deer (*Odocoileus virginianus*), axis deer (*Axis axis*), sika deer (*Cervus nippon*) and fallow deer (*Dama dama*) from Texas. J. Eukaryot. Microbiol. 46, 125–131. 10.1111/j.1550-7408.1999.tb04595.x 10361734

[B20] Dehority B. A. Odenyo A. A. (2003). Influence of diet on the rumen protozoal fauna of indigenous African wild ruminants. J. Eukaryot. Microbiol. 50, 220–223. 10.1111/j.1550-7408.2003.tb00121.x 12836880

[B21] Dogiel V. A. (1927). Monographie der Familie Ophryoscolecidae, Teil I. Arch. fur Protistenkd. 59, 1–289.

[B22] Duarte E. R. Abrão F. O. Oliveira Ribeiro I. C. Vieira E. A. Nigri A. C. Silva K. L.. (2018). Rumen protozoa of different ages of beef cattle raised in tropical pastures during the dry season. J. Appl. Anim. Res. 46, 1457–1461. 10.1080/09712119.2018.1530676

[B23] Eadie J. M. (1962). Inter-relationships between certain rumen ciliate protozoa. J. Gen. Microbiol. 29, 579–588. 10.1099/00221287-29-4-579

[B24] Eadie J. M. (1967). Studies on the ecology of certain rumen ciliate protozoa. J. Gen. Microbiol. 49, 175–194. 10.1099/00221287-49-2-175 4965673

[B25] Furman O. Shenhav L. Sasson G. Kokou F. Honig H. Jacoby S.. (2020). Stochasticity constrained by deterministic effects of diet and age drive rumen microbiome assembly dynamics. Nat. Commun. 11:1904. 10.1038/s41467-020-15652-8 32312972 PMC7170844

[B26] Gebczyńska Z. Gebczyński M. Martynowicz E. (1991). Food eaten by the free-living European bison in białowieza forest. Acta Theriol. 36, 307–313. 10.4098/AT.arch.91-32

[B27] Giesecke D. Gylswyk N. O. Van (1975). A study of feeding types and certain rumen functions in six species of South African wild ruminants. J. Agric. Sci. 85, 75–83. 10.1017/S0021859600053430

[B28] Guan L. L. Nkrumah J. D. Basarab J. A. Moore S. S. (2008). Linkage of microbial ecology to phenotype: correlation of rumen microbial ecology to cattle's feed efficiency. FEMS Microbiol. Lett. 288, 85–91. 10.1111/j.1574-6968.2008.01343.x 18785930

[B29] Henderson G. Cox F. Ganesh S. Jonker A. Young W. Global Rumen Census Collaborators G. R. C.. (2015). Rumen microbial community composition varies with diet and host, but a core microbiome is found across a wide geographical range. Sci. Rep. 5:14567. 10.1038/srep14567 26449758 PMC4598811

[B30] Horáková K. (1988). “Mikroskopické metódy (Microscopic methods),” in Mikrobiologické Laboratórne Metódy (Microbiological Laboratory Methods), eds V. Betina, H. Barátová, A. Fargašová, V. Frank, K. Horáková, and E. Šturdík (Bratislava: Alfa), 173–206.

[B31] Howard B. H. (1959). The biochemistry of rumen protozoa. 1. Carbohydrate fermentation by *Dasytricha* and *Isotricha*. Biochem. J. 71, 671–675. 10.1042/bj0710671 13651115 PMC1196856

[B32] Imai S. (1988). Ciliate protozoa in the rumen of Kenyan zebu cattle, Bos taurus indicus, with the description of four new species. J. Protozool. 35, 130–136. 10.1111/j.1550-7408.1988.tb04092.x 3130479

[B33] Imai S. Oku Y. Morita T. Ike K. Guirong (2004). Rumen ciliate protozoal fauna of reindeer in Inner Mongolia, China. J. Vet. Med. Sci. 66, 209–212. 10.1292/jvms.66.209 15031553

[B34] Ishaq S. L. Sundset M. A. Crouse J. Wright A.-D. G. (2015). High-throughput DNA sequencing of the moose rumen from different geographical locations reveals a core ruminal methanogenic archaeal diversity and a differential ciliate protozoal diversity. Microb. Genomics 1:e000034. 10.1099/mgen.0.000034 28348818 PMC5320624

[B35] Ito A. Imai S. (1998). Infraciliary bands in the rumen Ophryoscolecid ciliate *Ostracodinium* gracile (Dogiel, 1925), observed by light microscopy. J. Eukaryot. Microbiol. 45, 628–636. 10.1111/j.1550-7408.1998.tb04559.x 9864852

[B36] Ito A. Imai S. Manda M. Ogimoto K. (1995). Rumen ciliates of Tokara native goat in Kagoshima, Japan. J. Vet. Med. Sci. 57, 355–357. 10.1292/jvms.57.355 7492663

[B37] Ito A. Imai S. Ogimoto K. (1994). Rumen ciliate composition and diversity of Japanese beef black cattle in comparison with those of Holstein-Friesian cattle. J. Vet. Med. Sci. 56, 707–714. 10.1292/jvms.56.707 7999896

[B38] Ito A. Miyazaki Y. ImaI S. (2001). Light microscopic observations of infraciliature and morphogenesis in six species of rumen *Ostracodinium* ciliates. J. Eukaryot. Microbiol. 48, 440–448. 10.1111/j.1550-7408.2001.tb00177.x 11456320

[B39] Jost L. (2006). Entropy and diversity. Oikos 113, 363–375. 10.1111/j.2006.0030-1299.14714.x

[B40] Kamler J. (1999). Infusorial Concentration in Rumen Fluid of Red Deer, Fallow Deer, Roe Deer and Mouflon. Acta Vet. Brno 68, 247–252. 10.2754/avb199968040247

[B41] Kamler J. Dvorák J. Kamlerová K. (2003). Differences in relative volume and weight of forestomachs among four free living ruminants. Acta Vet. Brno 72, 33–39. 10.2754/avb200372010033

[B42] Karnati S. K. R. Yu Z. Sylvester J. T. Dehority B. A. Morrison M. Firkins J. L. (2003). Technical note: Specific PCR amplification of protozoal 18S rDNA sequences from DNA extracted from ruminal samples of cows. J. Anim. Sci. 81, 812–815. 10.2527/2003.813812x 12661662

[B43] Kittelmann S. Janssen P. H. (2011). Characterization of rumen ciliate community composition in domestic sheep, deer, and cattle, feeding on varying diets, by means of PCR-DGGE and clone libraries. FEMS Microbiol. Ecol. 75, 468–481. 10.1111/j.1574-6941.2010.01022.x 21204869

[B44] Kleynhans C. J. (1982). The rumen ciliates of greater kudu *Tragelaphus strepsiceros* (Pallas) from South Africa and Zimbabwe with a description of one new species. South African J. Zool. 17, 11–14. 10.1080/02541858.1982.11447771

[B45] Kleynhans C. J. van Hoven W. (1976). Rumen protozoa of the giraffe with a description of two new species. Afr. J. Ecol. 14, 203–214. 10.1111/j.1365-2028.1976.tb00164.x

[B46] Kofoid C. A. Christianson J. F. (1934). Ciliates from Bos gaurus H. Smith. Berkeley, CA: University of California Press.

[B47] Korchagina T. A. (2006). Taxonomic diversity of endobiotic ciliates in different parts of the elk' stomach (*Alces alces*). (in Russian). Omsk. Nauchnyj Vestn. 46, 244–246.

[B48] Korchagina T. A. (2012). Species diversity and population of ciliate endobionts in reindeer stomach (*Rangifer tarandus L*.). (in Russian). Perspekt. Nauk. 30, 5–10.

[B49] Kowalczyk R. Taberlet P. Coissac E. Valentini A. Miquel C. Kamiński T.. (2011). Influence of management practices on large herbivore diet—Case of European bison in Białowieza Primeval Forest (Poland). For. Ecol. Manage. 261, 821–828. 10.1016/j.foreco.2010.11.026

[B50] Kowalczyk R. Wójcik J. M. Taberlet P. Kamiński T. Miquel C. Valentini A.. (2019). Foraging plasticity allows a large herbivore to persist in a sheltering forest habitat: DNA metabarcoding diet analysis of the European bison. For. Ecol. Manage. 449:117474. 10.1016/j.foreco.2019.117474

[B51] Krasińska M. Krasiński Z. A. (2013). European Bison. The Nature Monograph. Berlin; Heidelberg: Springer. 10.1007/978-3-642-36555-3

[B52] Martinele I. D'Agosto M. (2008). Predação e canibalismo entre protozoários ciliados (*Ciliophora: Entodiniomorphida: Ophryoscolecidae*) no rúmen de ovinos (*Ovis aries*). Rev. Bras. Zool. 25, 451–455. 10.1590/S0101-81752008000300010

[B53] Michalowski T. Belzecki G. Kwiatkowska E. Pajak J. J. (2003). The effect of selected rumen fauna on fibrolytic enzyme activities, bacterial mass, fibre disappearance and fermentation pattern in sheep. J. Anim. Feed Sci. 12, 45–64. 10.22358/jafs/67642/2003

[B54] Mishima T. Katamoto H. Horii Y. Kakengi V. A. M. M. Ito A. (2009). Rumen ciliates from Tanzanian short horn zebu cattle, Bos taurus indicus, and the infraciliature of *Entodinium palmare n.sp*. and *Enoploplastron stokyi* (Buisson, 1924). Eur. J. Protistol. 45, 77–86. 10.1016/j.ejop.2008.07.002 19004625

[B55] Moon-van der Staay S. Y. Van der Staay G. W. M. M. Michalowski T. Jouany J.-P. P. Pristas P. Javorský P.. (2014). The symbiotic intestinal ciliates and the evolution of their hosts. Eur. J. Protistol. 50, 166–173. 10.1016/j.ejop.2014.01.004 24703617

[B56] Naga M. A. El-Shazly K. (1968). The metabolic characterization of the ciliate protozoon *Eudiplodinium medium* from the rumen of buffalo. J. Gen. Microbiol. 53, 305–315. 10.1099/00221287-53-3-305 4976557

[B57] Obidziński A. Miltko R. Bolibok L. Wajdzik M. Skubis J. Nasiadka P. (2017). Variation of natural diet of free ranging mouflon affects their ruminal protozoa composition. Small Rumin. Res. 157, 57–64. 10.1016/j.smallrumres.2017.09.019

[B58] Ogimoto K. Imai S. (1981). Atlas of Rumen Microbiology. Tokyo: Scientific Societies Press.

[B59] O'Kelly J. Spiers W. (1992). Possible contribution of protozoa to differences in rumen metabolism between cattle breeds. Aust. J. Agric. Res. 43:1795. 10.1071/AR9921795

[B60] Prins R. A. Geelen M. J. H. (1971). Rumen Characteristics of Red Deer, Fallow Deer, and Roe Deer. J. Wildl. Manage. 35:673. 10.2307/3799772

[B61] Przybyło M. Hummel J. Ortmann S. Codron D. Kohlschein G. M. Kilga D.. (2019). Digesta passage in nondomestic ruminants: separation mechanisms in “moose-type” and “cattle-type” species, and seemingly atypical browsers. Comp. Biochem. Physiol. A Mol. Integr. Physiol. 235, 180–192. 10.1016/j.cbpa.2019.06.010 31220621

[B62] Pucek Z. Belousova I. P. Krasiñska M. Krasiñski Z. A. Olech W. (2002). European bison, Bison bonasus: Current state of the Species and an Action Plan for its Conservation, ed Z. Pucek Bialowieza,: Mammal Research Institute, Polish Academy of Sciences. Available online at: https://ibs.bialowieza.pl/publications/1360.pdf (accessed May 30, 2021).

[B63] Purser D. B. Moir R. J. (1966a). Rumen volume as a factor involved in individual sheep differences. J. Anim. Sci. 25, 509–515. 10.2527/jas1966.252509x

[B64] Purser D. B. Moir R. J. (1966b). Variations in rumen volume and associated effects as factors influencing metabolism and protozoa concentrations in the rumen of sheep. J. Anim. Sci. 25, 516–520. 10.2527/jas1966.252516x 4961306

[B65] Reis C. C. dos Maeda E. M. Cedrola F. Martins E. N. Paula F. M. De Martinele I. (2019). Diet and breed alter community structures of rumen protozoa in cattle subjected to different feeding systems. Semin. Ciências Agrárias 40:909. 10.5433/1679-0359.2019v40n2p909

[B66] Robbins C. T. Spalinger D. E. van Hoven W. (1995). Adaptation of ruminants to browse and grass diets: are anatomical-based browser-grazer interpretations valid?. Oecologia 103, 208–213. 10.1007/BF00329082 28306775

[B67] Selinummi J. Seppälä J. Yli-Harja O. Puhakka J. A. (2005). Software for quantification of labeled bacteria from digital microscope images by automated image analysis. Biotechniques 39, 859–863. 10.2144/000112018 16382904

[B68] Shi P. J. Meng K. Zhou Z. G. Wang Y. R. Diao Q. Y. Yao B. (2008). The host species affects the microbial community in the goat rumen. Lett. Appl. Microbiol. 46, 132–135. 10.1111/j.1472-765X.2007.02274.x 17971095

[B69] Siritantikorn S. Jintaworn S. Noisakran S. Suputtamongkol Y. Paris D. H. Blacksell S. D. (2012). Application of ImageJ program to the enumeration of Orientia tsutsugamushi organisms cultured *in vitro*. Trans. R. Soc. Trop. Med. Hyg. 106, 632–635. 10.1016/j.trstmh.2012.05.004 22789674 PMC3449237

[B70] Sládeček F. (1946). Ophryoscolecidae z bachoru jelena (*Cervus elaphus* L.), danka (*Dama dama* L.) a srnce (*Capreolus* L.). Ophryoscolecidae from the stomach of *Cervus elaphus* L., *Dama dama* L., and *Capreolus capreolus* L. Věstník Csl. Zool. společnosti 10, 201–231.

[B71] Spellerberg I. F. Fedor P. J. (2003). A tribute to Claude Shannon (1916-2001) and a plea for more rigorous use of species richness, species diversity and the ‘shannon-wiener’ index. Glob. Ecol. Biogeogr. 12, 177–179. 10.1046/j.1466-822X.2003.00015.x

[B72] Tan C. Ramírez-Restrepo C. A. Shah A. M. Hu R. Bell M. Wang Z.. (2020). The community structure and microbial linkage of rumen protozoa and methanogens in response to the addition of tea seed saponins in the diet of beef cattle. J. Anim. Sci. Biotechnol. 11:80. 10.1186/s40104-020-00491-w 32832076 PMC7422560

[B73] Tapio I. Fischer D. Blasco L. Tapio M. Wallace R. J. Bayat A. R.. (2017). Taxon abundance, diversity, co-occurrence and network analysis of the ruminal microbiota in response to dietary changes in dairy cows. PLoS ONE 12:e0180260. 10.1371/journal.pone.0180260 28704445 PMC5509137

[B74] Towne G. Nagaraja T. G. Cochran R. C. Harmon D. L. Owensby C. E. Kaufman D. W. (1988a). Comparisons of ruminal fermentation characteristics and microbial populations in bison and cattle. Appl. Environ. Microbiol. 54, 2510–2514. 10.1128/AEM.54.10.2510-2514.1988 3272131 PMC204300

[B75] Towne G. Nagaraja T. G. Kemp K. K. (1988b). Ruminal ciliated protozoa in bison. Appl. Environ. Microbiol. 54, 2733–2736. 10.1128/AEM.54.11.2733-2736.1988 3145709 PMC204364

[B76] Williams A. G. (1986). Rumen holotrich ciliate protozoa. Microbiol. Rev. 50, 25–49. 10.1128/MMBR.50.1.25-49.1986 3083220 PMC373052

[B77] Williams A. G. Coleman G. S. (1992). The Rumen Protozoa. New York, NY: Springer New York. 10.1007/978-1-4612-2776-2

[B78] Williams C. L. Thomas B. J. McEwan N. R. Rees Stevens P. Creevey C. J. Huws S. A. (2020). Rumen protozoa play a significant role in fungal predation and plant carbohydrate breakdown. Front. Microbiol. 11:720. 10.3389/fmicb.2020.00720 32411103 PMC7200989

[B79] Xue M. Y. Sun H. Z. Wu X. H. Liu J. X. Guan L. L. (2020). Multi-omics reveals that the rumen microbiome and its metabolome together with the host metabolome contribute to individualized dairy cow performance. Microbiome 8:64. 10.1186/s40168-020-00819-8 32398126 PMC7218573

[B80] Zeitz J. O. Amelchanka S. L. Michałowski T. Wereszka K. Meile L. Hartnack S.. (2012). Effect of the rumen ciliates *Entodinium caudatum, Epidinium ecaudatum* and *Eudiplodinium maggii*, and combinations thereof, on ruminal fermentation and total tract digestion in sheep. Arch. Anim. Nutr. 66, 180–199. 10.1080/1745039X.2012.676817 22724165

